# A New Method Combining Pattern Prediction and Preference Prediction for Next Basket Recommendation

**DOI:** 10.3390/e23111430

**Published:** 2021-10-29

**Authors:** Guisheng Chen, Zhanshan Li

**Affiliations:** 1 College of Computer Science and Technology, Jilin University, Changchun 130012, China; chengs16@mail.jlu.edu.cn; 2 Key Laboratory of Symbolic Computation and Knowledge Engineering, Ministry of Education, Jilin University, Changchun 130012, China

**Keywords:** recommendation systems, market basket recommendation, data mining, periodic pattern, sequential rule, association rule

## Abstract

Market basket prediction, which is the basis of product recommendation systems, is the concept of predicting what customers will buy in the next shopping basket based on analysis of their historical shopping records. Although product recommendation systems develop rapidly and have good performance in practice, state-of-the-art algorithms still have plenty of room for improvement. In this paper, we propose a new algorithm combining pattern prediction and preference prediction. In pattern prediction, sequential rules, periodic patterns and association rules are mined and probability models are established based on their statistical characteristics, e.g., the distribution of periods of a periodic pattern, to make a more precise prediction. Products that have a higher probability will have priority to be recommended. If the quantity of recommended products is insufficient, then we make a preference prediction to select more products. Preference prediction is based on the frequency and tendency of products that appear in customers’ individual shopping records, where tendency is a new concept to reflect the evolution of customers’ shopping preferences. Experiments show that our algorithm outperforms those of the baseline methods and state-of-the-art methods on three of four real-world transaction sequence datasets.

## 1. Introduction

Data mining technology is an efficient tool for business. Early in 1993, Agrawal et al. [1] proposed association rule mining for transaction databases to discover the intrinsic connection between different products and the shopping habits of customers. This technology makes sales prediction easier. By predicting what customers will buy in the next shopping basket and then recommending the products to them, retailers can improve their services and promote sales. We call such a technique market basket prediction, which is the basis of product recommendation systems.

Since Agrawal et al. proposed association rule mining, both data mining and recommendation systems have been developing rapidly. On the one hand, sequential patterns [2], sequential rules [3], coverage patterns [4], temporal patterns [5], subgraph patterns [6] and periodic patterns [7] have been proposed. Data mining, as an increasingly sophisticated technology, has been used for many domains, such as time series analysis [8], medicine [9] and image processing [10]. On the other hand, recommendation systems include other kinds of implementation methods including pattern-based models, collaborative filtering [11] and Markov chains [12]. Advances in data mining technology make pattern-based models promising. There are many efficient and ready-made algorithms for pattern mining [13], and they can be easily used to implement pattern-based recommendation systems.

A pattern reveals the relation between different products, which makes pattern-based models comprehensive. Among them, a sequential rule reveals the relation between the products in two consecutive transactions. This means that a customer bought a product at some time and will buy another product at a future time. For example, if a customer buys a computer, he or she will need a U-Disk or printer possibly when working on the computer. The higher the confidence of a sequential rule, the higher the possibility. A number of sophisticated and efficient sequential rule mining algorithms have been proposed, including RuleGen [13], ERMiner [14] and RuleGrowth [3,15]. Because all products have a limited service life, when a product is used out, we will buy another product again. Therefore, some products periodically appear in our market baskets. If we know the period of a periodic pattern, then we can predict when it will appear again. Some periodic pattern mining algorithms have been proposed, including SPP [16], MPFPS [17] and LPPM [18]. The association rule reveals the relation between the products in a basket. This means that if a customer buys a product, he or she will buy another product at the same time. Some efficient algorithms such as TopKRules [19] and TNR [20] focus on association rule mining.

As described above, pattern-based models have the advantages of popularity and comprehensibility. However, existing pattern-based recommendation algorithms are insufficient to capture customers’ shopping habits. For example, Ref. [21] focused on the association rule only, and the periodicity was neglected. Furthermore, the statistical characteristics inside a pattern, e.g., the distribution of periods of a periodic pattern, are also neglected. For example, by the current definition of periodic patterns [22], when a periodic pattern is used to make a prediction, we can only predict that the pattern will reoccur between a time interval but the probability at an exact time.

In terms of the disadvantages of existing methods, our method leverages not only the association rule but also the sequential rule and periodic pattern for prediction at the same time. We call such a strategy pattern prediction. Furthermore, the frequency and tendency of a product will be considered the preference that customers have for this product and the evolution of preference, respectively, to make predictions, which we call preference prediction. Combining pattern prediction and preference prediction, we propose a new algorithm for market basket prediction, which we call SPAP (Sequential rule, Periodic pattern, Association rule, and Preference).

In this paper, first, we present a new definition of periodic patterns and tendencies. Generally, if a product is bought periodically by a customer, then the period will be nearly equal to the service life of the product. However, service lives of a kind of product may differ from one another, leading to a fluctuation in the period. What type of pattern is a periodic pattern, which has a virtue to reveal the periodicity of product purchases that have not a fixed period? Obviously, if a pattern has periods that the fluctuation is too large compared to the average period, it will not be periodic. Taking the average period and standard deviation into account, the coefficient of variation, which is the specific value of the standard deviation and mean value, is used to measure the periodicity of patterns in our definition. The concept of tendency is based on the following considerations: if a product is more frequently bought in recent baskets than in early baskets of a customer, then the customer tends to be increasingly inclined to the product. Otherwise, if a product is more frequently bought in early baskets than recent ones, the customer tends to be increasingly estranged from the product. We use a new concept of tendency to reflect this fact.

Second, we propose probability models for pattern prediction. The sequential rule reveals the relation of two patterns belonging to two consecutive transactions. The former pattern is called the antecedent, and the latter the consequent. When a sequential rule is used for predicting the next basket, the time interval between antecedent and consequent is usually neglected. That is, the consequent will follow the antecedent with a given confidence, however, we do not know for sure at what time it occurs. In this paper, we learn the statistical model for the time interval of all sequential rules in the training data. In prediction, we use the statistical model to compute the probability of the occurrence of consequents at an exact time stamp. For periodic patterns, the statistical model is determined by the average period and standard deviation. After training, we obtain all sequential rules, periodic patterns and association rules, along with their statistical characteristics. Consequently, we can calculate the probability of all products in a customer’s next basket. Products that have a higher probability will have priority to be recommended. If the quantity of recommended products is insufficient, then we will make a preference prediction to select more products.

Preference prediction is based on this observation: if a product is more frequently bought by a customer, then we draw the conclusion that the customer has a preference for this product, and this product will have priority to be recommended. If some products have the same frequency, then the product with a higher tendency will be selected first in such a case.

Our contributions in this paper are summarized as follows:

• We present a new definition for periodic patterns and the tendency of patterns.
• We propose probability models for pattern prediction to predict the next basket.
• We design a new algorithm combining pattern prediction and preference prediction for next basket recommendation.
• Empirically, we show that our algorithm outperforms the baseline methods and state-of-the-art methods on three of four real-world transaction sequence datasets under the evaluation metrics of F1-Score and Hit-Ratio.

The remainder of this paper is organized as follows: Section 2 reviews the existing approaches. Section 3 includes the preliminary. We introduce our prediction method in Section 4. The implementation of our algorithm is described in Section 5, and the experimental analysis is reported in Section 6. Finally, we draw a conclusion in Section 7.

## 2. Related Work

Implementation methods of recommendation systems can be categorized into sequential, general, pattern-based, and hybrid models. Sequential models [23,24], mostly relying on Markov chains, explore sequential transaction data by predicting the next purchase based on the last actions to capture sequential behavior. A major advantage of this model is its ability to capture sequential behavior to provide good recommendations. The general model [25], in contrast, does not consider sequential behavior but makes recommendations based on customers’ whole purchase history. The key idea is collaborative filtering. The pattern-based model bases predictions on the frequent patterns that are extracted from the shopping records of all customers [26]. Among them, the hybrid model combines the models mentioned above or other ideas, such as graph-based models [27] and recurrent neural network models [28,29]. Since there are so many works devoted to recommendation systems, it is impossible to list all here. So, we only briefly review pattern-based approaches in the next paragraph.

Fu et al. [30] first used an association rule for recommendation systems. Candidate items are listed for her in their order of support. Wang et al. [31] proposed an association rule mining algorithm with maximal nonblank for recommendation. The weighted association rule mining algorithm based on FP-tree and its application procedure in personalization recommendation was given by Wang et al. [32]. Ding et al. [33,34] proposed a method for personalized recommendation, which could decrease the number of association rules by merging different rules. Li et al. [21,35] proposed the notion of strongest association rules (SARs), and developed a matrix-based algorithm for mining SAR sets. As the subset of the entire association rule set, the SAR set includes many fewer rules with the special suitable form for personalized recommendation without information loss. Lazcorreta et al. [26] applied a modified version of the well-known apriori data mining algorithm towards personalized recommendation. Najafabadi et al. [36] applied the users’ implicit interaction records with items to efficiently process massive data by employing association rules mining. It captures the multiple purchases per transaction in association rules, rather than just counting total purchases made. Chen et al. [37] mined simple association rules with a single item in consequent to avoid exponential pattern growth. The method proposed by Zhou et al. [38] involved implementation of genetic network programming and ant colony optimization to solve the sequential rule mining problem for commercial recommendations in time-related transaction sequence databases. Maske et al. [39] proposed a method describing how customer behavior predicted based on the customer purchase items by association rule mining algorithm Apriori.

In the other methods, Cumby et al. [40] proposed a predictor that embraces a user-centric vision by reformulating basket prediction as a classification problem. They build a distinct classifier for every customer and perform predictions by relying just on their personal data. Unfortunately, this approach assumes the independence of items purchased together. Wang et al. [41] employed a two-layer structure to construct a hybrid representation over customers and items purchase history from last transactions: the first layer represents the transactions by aggregating item vectors from the last transactions, while the second layer realizes the hybrid representation by aggregating the customer’s vectors and the transactions representations. Guidotti et al. [42,43,44] defined a new pattern named the Temporal Annotated Recurring Sequence (TARS), which seeks to simultaneously and adaptively capture the co-occurrence, sequentiality, periodicity and recurrence of the items in the transaction sequence. Jain et al. [45] designed a business strategy prediction system for market basket analysis. Kraus et al. [46] proposed similarity matching based on subsequential dynamic time warping as a novel predictor of market baskets, and leverage the Wasserstein distance for measuring the similarity among embedded purchase histories. Hu et al. [47] presented a k-nearest neighbors (kNN) based method to directly capture two useful patterns: repeated purchase pattern and collaborative purchase pattern that associate with personalized item frequency. Faggioli et al. [48] proposed an efficient solution to achieve the next basket recommendation, under a more general top-n recommendation framework by exploiting a set of collaborative filtering based techniques to capture customers’ shopping patterns and intentions.

## 3. Preliminary

Retailers usually preserve their customers’ shopping histories in a database that we call the transaction sequence database. A customer’s shopping history contains many transactions. Transaction, also called basket, usually contains ID, date, product list and quantity. All transactions of a customer are sorted according to date. This is called the transaction sequence, as Table 1 shows. A transaction sequence database contains all customers’ transaction sequence, as Table 2 shows.

Let $C=\{c_{1},...,c_{n}\}$ be a set of *n* customers and $I=\{i_{1},...,i_{m}\}$ be a set of *m* items or products in the market. The transaction sequence of customer *c* is denoted as $B_{c}$, and $B_{c}=\langle b_{1},...,b_{r_{c}}\rangle$, where $b_{i}\subseteq I$, $i\in \{1,...,r_{c}\}$, denotes a transaction or basket. The terms transaction, basket and itemset will be used interchangeably, due to the fact that we are referring to an unordered set of items (or products). The size of sequence $B_{c}$ is denoted as $|B_{c}|$, and $|B_{c}|=r_{c}$. $b_{r_{c}+1}$ denotes the next basket that will be purchased by customer *c* at the next time. We use indexes set $\left\{1,...,r_{c}\right\}$ of baskets in transaction sequence rather than formal dates as timestamps to simplify the problem. The interval of two transactions is denoted as $Gap(b_{i},b_{j})$ and defined as $Gap(b_{i},b_{j})=j-i$, where $1\leq i<j\leq r_{c}$. The transaction sequence dataset $D=\{B_{c_{1}},...,B_{c_{n}}\}$ consists of transaction sequences of *n* customers.

**Problem** **1.** *Assume a transaction sequence dataset, our aim is to predict the next basket for each customer according to their transaction sequence. Then, we will select k products to recommend to him or her. Formally, given dataset D, for all transaction sequence* $B_{c}\in D$ *and* $B_{c}=\langle b_{1},...,b_{r_{c}}\rangle$ *to predict* $b_{r_{c}+1}$ *, which contains a set of candidate items for recommendation. Let* $b_{c}^{*}$ *denote the selected item set; then,* $b_{c}^{*}$ *contains k items selected from* $b_{r_{c}+1}$ *to recommend to customer c.*

**Definition** **1.** *(Frequent Itemset Pattern) Given an itemset p,* p⊆I *, frequency threshold θ, and transaction sequence* $B=\langle b_{1},...,b_{r}\rangle$ *. If* $\exists b_{i}\in B$ *, we have* $p\subseteq b_{i}$ *, then we call* $b_{i}$ *a support for p. Let* Sup(p) *denotes all supports of p, then* $Sup\left(p\right)=\{b_{i}|\forall b_{i}\in B,$ $p\subseteq b_{i}\}$ *. The absolute frequency of p is defined as* Freq(p)=|Sup(p)| *, and the relative frequency is* Freq(p)=|Sup(p)|/|B| *. If* Freq(p)≥θ *, then we call p a frequent itemset pattern and itemset pattern for short. If p contains only a single item, that is,* |p|=1 *, we call it a single item pattern.*

**Definition** **2.** *(Association Rule) Given two itemset patterns* $p_{1}$ *and* $p_{2}$ *of a transaction sequence, confidence threshold η, if*

(1)
$p_{1}\neq ⌀,p_{2}\neq ⌀$ and $p_{1}\cap p_{2}=⌀$ *,*
(2)
$p=p_{1}\cup p_{2}$ *is an itemset pattern, and*
(3)
$Freq\left(p\right)/Freq\left(p_{1}\right)\geq \eta$ *,*

*then* $p_{1}\to p_{2}$ *is an association rule. Its frequency is denoted as* $Freq(p_{1}\to p_{2})$ *and defined as* $Freq\left(p_{1}\to p_{2}\right)=Freq\left(p\right)$ *. Its confidence is denoted as* $Conf(p_{1}\to p_{2})$ *and defined as* $Conf\left(p_{1}\to p_{2}\right)=Freq\left(p\right)/Freq\left(p_{1}\right)$ *. We call* $p_{1}$ *the antecedent, and* $p_{2}$ *the consequent.*

**Definition** **3.** *(Frequent Sequential Pattern) A sequence* $s=\langle p_{1},...,p_{k}\rangle$ *is a subsequence of transaction sequence* $B=\langle b_{1},...,b_{r}\rangle$ *, denoted as* s≺B *, if and only if there exist k integers* $\left\{e_{1},...,e_{k}\right\}$ *such that* k≤r *,* $e_{1}<\cdots <e_{k}$ *and* ∀i∈{1,...,k} *it holds that* $p_{i}\subseteq b_{e_{i}}$ *. We call this integer set an embedding of s in B, denoted as* Emb(s) *,* $Emb\left(s\right)=\left\{e_{1},...,e_{k}\right\}$ *.* Embs(s) *denotes the set of all embeddings of s in B. In transaction sequence dataset D, if* $s≺B_{c}$ *, we call* $B_{c}$ *a support of s. The set of all supports of s is denoted as* Sup(s) *and* $Sup\left(s\right)=\{B_{c}|\forall B_{c}\in D,$ $s≺B_{c}\}$ *. The absolute frequency of s is defined as* Freq(s)=|Sup(s)| *and the relative frequency* Freq(s)=|Sup(s)|/|D| *. Given a threshold θ for frequency, if* Freq(s)≥θ *, then s is a frequent sequential pattern, sequential pattern for short.*

In this paper, the length of *s* is denoted as Len(s), and defined as $Len\left(s\right)=\sum_{v\in \{1,...,k\}}\left|p_{v}\right|$. If a sequence *s* contains only an itemset *p*, that is, s=〈p〉, |s|=1, then it can be mapped into itemset *p*, and we have s=p. If Len(s)=1, viz. *s* has only a single itemset, and this itemset contains only a single item. We call it a single item pattern.

**Definition** **4.** *(Sequential Rule) Given two sequential patterns* $s_{1}$ *and* $s_{2}$ *of a transaction sequence dataset and confidence threshold η, if*

(1)
$s_{1}\neq ⌀,s_{2}\neq ⌀$ *,*
(2)
$s=\langle s_{1},s_{2}\rangle$ *(* $s_{1}$ *concatenates with* $s_{2}$ *) is a sequential pattern, and*
(3)
$Freq\left(s\right)/Freq\left(s_{1}\right)\geq \eta$ *,*

*then we call* $s_{1}\to s_{2}$ *a sequential rule. Its frequency* $Freq\left(s_{1}\to s_{2}\right)=Freq\left(s\right)$ *, and confidence* $Conf\left(s_{1}\to s_{2}\right)=Freq\left(s\right)/Freq\left(s_{1}\right)$ *. We call* $s_{1}$ *the antecedent, and* $s_{2}$ *the consequent.*

**Property** **1.** *Assume a sequential rule* $s_{1}\to s_{2}:Conf$ *, where* $s_{2}=\langle p_{1},...,p_{k}\rangle$ *, then we have* $s_{1}\to \langle p_{1}\rangle :Conf_{1}$ *,...,* $s_{1}\to \langle p_{k}\rangle :Conf_{k}$ *, and* $Conf\leq Conf_{1}$ *,...,* $Conf\leq Conf_{k}$ *.*

**Proof.**  The proof comes from the anti-monotonicity of sequential patterns’ frequency.    □

**Definition** **5.** *(Periodic Pattern) Let p be an itemset pattern of transaction sequence* $B=\langle b_{1},...,b_{r}\rangle$ *,* λ=Freq(p) *. The occurrence list of p is denoted as* Occur(p) *, and defined as* Occur(p)={i|∀i∈{1,...,r} s.t. $b_{i}\in B$ and $p\subseteq b_{i}\}$ *. Then, the period list of p in B is denoted as* Per(p) *, and defined as* $Per\left(p\right)=\{per_{a}=w_{a+1}-w_{a}|\forall a\in \left\{1,...,\lambda -1\right\},$ $w_{a}\in Occur\left(p\right)\}$ *. The coefficient of variation of p is denoted as* Coefva(p) *, and* Coefva(p)=std(Per(p))/mean(Per(p)) *, where* std(*) *and* mean(*) *are the standard deviation and mean, respectively. Given a threshold δ for the coefficient of variation, if* Coefva(p)≤δ *, then p is a periodic itemset pattern or periodic pattern for short.*

**Definition** **6.** *(Gap of Two Itemsets) Given two itemsets* $p_{1}$ *and* $p_{2}$ *of transaction sequence B. If* $\exists o_{1}\in Occur\left(p_{1}\right),\exists o_{2}\in Occur\left(p_{2}\right)$ *and* $o_{1}<o_{2}$ *, then the gap of* $p_{1}$ *and* $p_{2}$ *in B is defined as* $Gap\left(p_{1},p_{2}\right)=min\left\{g|\forall o_{1}\in Occur\left(p_{1}\right),\forall o_{2}\in Occur\left(p_{2}\right),o_{1}<o_{2},g=o_{2}-o_{1}\right\}$ *; otherwise,* $Gap(p_{1},p_{2})=\infty$ *.*

**Definition** **7.** *(Gap of Two Subsequences) Given two subsequences* $s_{1}$ *and* $s_{2}$ *of transaction sequence B. If* $\exists E_{1}\in Embs\left(s_{1}\right),\exists E_{2}\in Embs\left(s_{2}\right)$ *and* $max\left(E_{1}\right)<min\left(E_{2}\right)$ *, then the gap of* $s_{1}$ *and* $s_{2}$ *in B is defined as* $Gap\left(s_{1},s_{2}\right)=min\left\{g\right|\forall E_{1}\in Embs\left(s_{1}\right),\forall E_{2}\in Embs\left(s_{2}\right),$ $max\left(E_{1}\right)<min\left(E_{2}\right),$ $g=min\left(E_{2}\right)-max\left(E_{1}\right)\}$ *; otherwise,* $Gap(p_{1},p_{2})=\infty$ *.*

**Definition** **8.** *(Tendency) Given an itemset p of transaction sequence B, we call* Ten(p)=mean(Occur(p)) *the tendency of itemset p in transaction sequence B.*

**Example** **1.** *For the transaction sequence given in* Table 1 *, the support of itemset* p={a,h} *is* $Sup\left(p\right)=\left\{b_{3},b_{9},b_{11}\right\}$ *, frequency* Freq(p)=3 *,* Occur(p)={3,9,11} *,* $per_{1}=9-3=6$ *,* $per_{2}=11-9=2$ *, periods list* Per(p)={6,2} *,* Coefva(p)=0.71 *. In* Table 2 *, the frequency of sequence* $s_{1}=\langle \left\{a,c\right\}\rangle$ *is* $Freq(s_{1})=7$ *, the frequency of sequence* $s_{2}=\langle \left\{f,g\right\}\rangle$ *is* $Freq(s_{2})=6$ *, and the frequency of sequence* $s=\langle s_{1},s_{2}\rangle$ *is* Freq(s)=6 *. We have* $Conf(s_{1}\to s_{2})=6/7$ *.* $Embs{\left(s\right)}_{c_{1}}=\left\{\left\{1,3\right\}\right\}$ *.* $Embs{\left(s\right)}_{c_{6}}=\left\{\left\{1,3\right\},\left\{1,4\right\},\left\{2,3\right\},\left\{2,4\right\}\right\}$ *. The frequencies of itemsets* $p_{1}=\left\{a,c\right\}$ *and* $p_{2}=\left\{f,g\right\}$ *in transaction sequence* $B_{c_{6}}$ *are* $Freq{\left(p_{1}\right)}_{c_{6}}=2$ *and* $Freq{\left(p_{2}\right)}_{c_{6}}=2$ *, respectively. The occurrence list of itemset* {d} *in transaction sequence* $B_{c_{2}}$ *is* $Occur{\left(\left\{d\right\}\right)}_{c_{2}}=\left\{1,4\right\}$ *, tendency* $Ten{\left(\left\{d\right\}\right)}_{c_{2}}=2.5$ *.*

## 4. Framework

Our method includes pattern prediction and preference prediction. In pattern prediction, first, all sequential rules, periodic patterns and association rules are found together with their statistical characteristics. Then, probability models are built based on their statistical characteristics. Afterward, we use the probability models to calculate the probability of all products in the next basket for a customer. The products that have a higher probability will be selected preferentially to recommend to him or her. If *k* products have been selected, then continue to the prediction of the next customer; otherwise, make preference predictions. In preference prediction, the product that is more frequent in the individual shopping records will be selected first. If some products have the same frequency, then the product that has a higher tendency will be selected. Until all *k* products are selected, we continue to predict the next customer. See Algorithm 1. We introduce the probability list pl to preserve the probability of all items in $b_{r+1}$, viz. pl:={(item1:value1),(item2:value2),...}.
**Algorithm 1:** SPAP$\left(D,\theta_{r},\eta ,\theta_{p},\delta ,k\right)$.
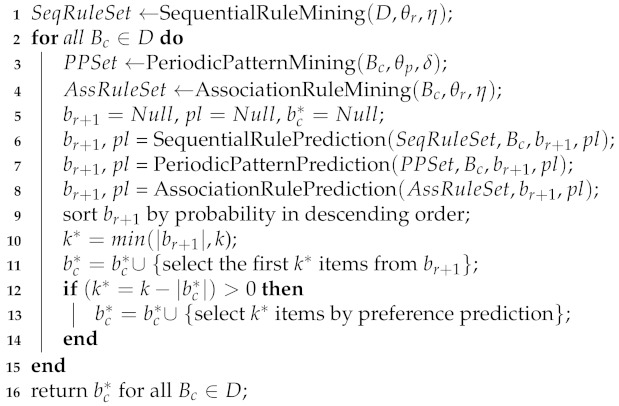


### 4.1. Sequential Rule Prediction

#### 4.1.1. Probability Model of Sequential Rule

Sequential rules reveal the relation between products in two consecutive transactions. This means that a customer bought a product at some time, and he or she will buy another product at a future time. However, the sequential rule defined by the previous section has a limitation to use for the next basket prediction. For example, given sequential rule $E_{1}\to E_{2}:Conf=0.8$. If event $E_{1}$ occurs, then it will lead to event $E_{2}$ occurring at a confidence of 0.8, and the confidence is considered as the probability here, that is, $P(E_{2}|E_{1})=0.8$. However, we did not know for sure at what time event $E_{2}$ occurs, and did not know the probability of event $E_{2}$ occurring at an exact time after event $E_{1}$ occurred. To address this limitation, we build a probability model for time intervals of the sequential rule. The time interval of a sequential rule, i.e., the time interval between event $E_{1}$ and event $E_{2}$, is a random variable and represented by *X* here, $X=Gap(E_{1},E_{2})$. Generally, the larger the time interval, the lower the relevance of $E_{1}$ and $E_{2}$, and vice versa. We suppose that the probability model nearly follows an exponential distribution with a parameter of $mean(Gap\left(s_{1},s_{2}\right))$.

**Example** **2.** *For a transaction sequence dataset showed in* Table 2 *, let* $s_{1}=\langle \left\{a,c\right\}\rangle$ *,* $s_{2}=\langle \left\{f,g\right\}\rangle$ *and* $s=\langle s_{1},s_{2}\rangle =\langle \left\{a,c\right\},\left\{f,g\right\}\rangle$ *, we have* $Sup\left(s_{1}\right)=\left\{c_{1},c_{2},c_{3},c_{4},c_{5},c_{6},c_{7}\right\}$ *,* $Sup\left(s_{2}\right)=\left\{c_{1},c_{2},c_{3},c_{4},c_{5},c_{6}\right\}$ *. If we set* θ=5 *,* η=0.8 *, and we have* $Conf(s_{1}\to s_{2})=6/7>\eta$ *, then* $s_{1}\to s_{2}$ *is a sequential rule. The time interval between* $s_{1}$ *and* $s_{2}$ *is* $Gap{\left(s_{1},s_{2}\right)}_{c_{1}}=3-1=2$ *in* $c_{1}$ *, where* $Occur{\left(s_{1}\right)}_{c_{1}}=\left\{1\right\}$ *and* $Occur{\left(s_{2}\right)}_{c_{1}}=\left\{3\right\}$ *. In a similar way,* $Gap{\left(s_{1},s_{2}\right)}_{c_{2}}=1$ *,* $Gap{\left(s_{1},s_{2}\right)}_{c_{3}}=1$ *,* $Gap{\left(s_{1},s_{2}\right)}_{c_{4}}=3$ *,* $Gap{\left(s_{1},s_{2}\right)}_{c_{5}}=2$ *,* $Gap{\left(s_{1},s_{2}\right)}_{c_{6}}=1$ *. Note that in sequence* $c_{6}$ *, we have* $Occur{\left(s_{1}\right)}_{c_{6}}=\left\{1,2\right\}$ *and* $Occur{\left(s_{2}\right)}_{c_{6}}=\left\{3,4\right\}$ *, leading* $Gap{\left(s_{1},s_{2}\right)}_{c_{6}}$ *to be multiple-valued. According to Definition 6, we have* $Gap{\left(s_{1},s_{2}\right)}_{c_{6}}=min\{\left(3-1\right),\left(3-2\right),$ (4−1), (4−2)}=1 *. Consequently, we obtain a probability model for* $Gap(s_{1},s_{2})$ *as* $P\{Gap\left(s_{1},s_{2}\right)=1\}=1/2$ *,* $P\{Gap\left(s_{1},s_{2}\right)=2\}=1/3$ *and* $P\{Gap\left(s_{1},s_{2}\right)=3\}=1/6$ *, respectively.*

#### 4.1.2. Principle of Sequential Rule Prediction

Given transaction sequence $B=\langle b_{1},...,b_{r}\rangle$, sequential rule $s_{1}\to s_{2}:Conf$, and its probability distribution $P\{X=Gap\left(s_{1},s_{2}\right)\}$ of time intervals between $s_{1}$ and $s_{2}$. Suppose the consequent contains only a single itemset, that is, $|s_{2}|=1$ (Since if $|s_{2}|>1$, then we can break it down into several sequential rules, which have a consequent containing only a single itemset, according to Property 1). If $s_{1}⊀B$, then $P(s_{1}≺B)=0$. Otherwise, $P(s_{1}≺\langle b_{1},...,b_{\lambda }\rangle )=1$, where $\lambda =max\{e|\forall E\in Embs\left(s_{1}\right),e\in E\}$. Since sequential rule $s_{1}\to s_{2}:Conf$ means that $P(s_{2}≺\langle b_{\lambda +1},b_{\lambda +2},...\rangle |s_{1}≺\langle b_{1},...,b_{\lambda }\rangle )=Conf$, then $P\left(s_{2}≺\langle b_{\lambda +1},b_{\lambda +2},...\rangle \right)=Conf\times P\left(s_{1}≺\langle b_{1},...,b_{\lambda }\rangle \right)=Conf$. Suppose $A_{1}$ denote the event $s_{2}\subseteq b_{r+1}$. In a similar way, $A_{u}$ denote the event $s_{2}\subseteq b_{r+u}$. If event $A_{1}$ occurs, then $Gap(s_{1},s_{2})=r+1-\lambda$, $P\left(A_{1}|s_{2}≺\langle b_{\lambda +1},b_{\lambda +2},...\rangle \right)=P\left\{X=\left(r+1-\lambda \right)\right\}$. In a similar way, if event $A_{u}$ occurs, then $Gap(s_{1},s_{2})=r+u-\lambda$, $P\left(A_{u}|s_{2}≺\langle b_{\lambda +1},b_{\lambda +2},...\rangle \right)=P\left\{X=\left(r+u-\lambda \right)\right\}$. Since $P(s_{2}≺\langle b_{\lambda +1},b_{\lambda +2},...\rangle )=Conf$, we obtain the probability of $s_{2}\subseteq b_{r+u}$, $P\left(A_{u}\right)=P\left\{X=\left(r+u-\lambda \right)\right\}\times Conf$.

First, the time variable is continuous in general. However, in our case, we use the index of baskets as the timestamp, so the time variable is discretized. The probability of an exact value of the time variable is the probability within a unit interval over this value. Second, if an item is contained in the consequents of several sequential rules, then it will be predicted several times. In such a case, we update its probability as the maximal value. See Algorithm 2.
**Algorithm 2:** SequentialRulePrediction$\left(RuleSet,B,b_{r+1},pl\right)$.
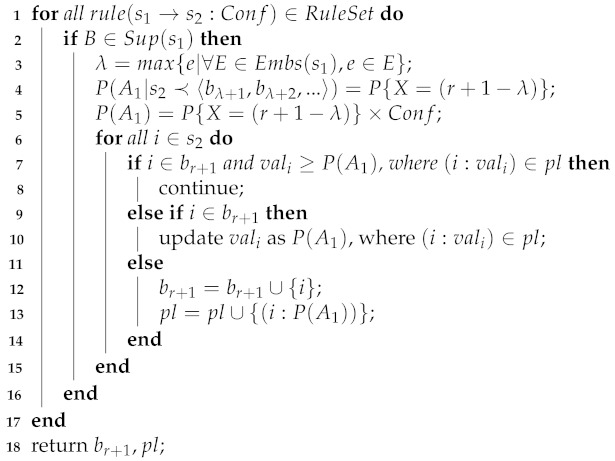


**Figure 1:**
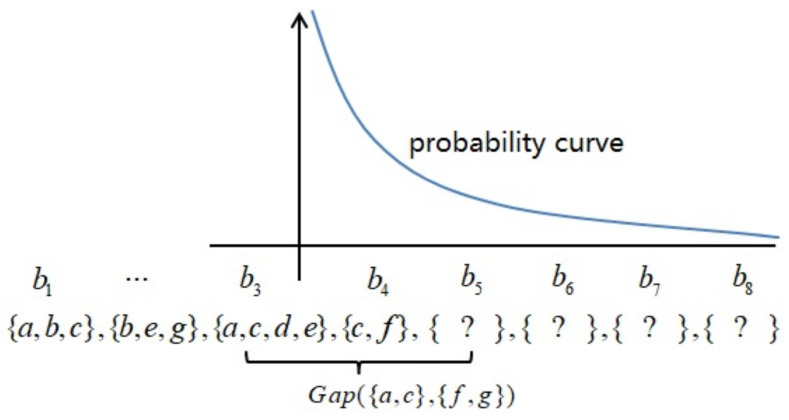
Probability model of sequential rule.

**Continue with the Example 2** Let B=〈{a,b,c},{b,e,g},{a,c,d,e},{c,f}〉; we predict the probability of all products in $b_{5}$. In Example 2, we obtain a sequential rule $s_{1}\to s_{2}:Conf$ and its probability model. $Embs\left(s_{1}\right)=\left\{\left\{1\right\},\left\{3\right\}\right\}$, λ=3. If $s_{2}\subseteq b_{5}$, then $Gap(s_{1},s_{2})=2$. We have $P\{Gap\left(s_{1},s_{2}\right)=2\}=1/3$ from the probability distribution. Finally, we have $P(\left\{f,g\right\}\subseteq b_{5})=6/7\times 1/3=6/21$, as Figure 1 shows.

### 4.2. Periodic Pattern Prediction

#### 4.2.1. Probability Model of Periodic Pattern

Periodic events intrinsically reoccur with a fixed period. However, in the real world, the period is influenced by different factors, leading to a fluctuation in the period. In this case, we only have an average value for periods. As we all know, the smaller the fluctuation, the better. So, we compare the fluctuation with the average period. If the fluctuation is too large compared to the average period, we do not define it as a periodic event. The coefficient of variation, which is the specific value of the standard deviation and mean value, is suitable for periodicity measure, since standard deviation is a good measure for fluctuation. The service life of a product is no exception. If a product is bought periodically by a customer, then the period will be nearly equal to the service life of the product. However, service lives of a kind of products may differ from one another, leading to a fluctuation in the period.

**Figure 2:**
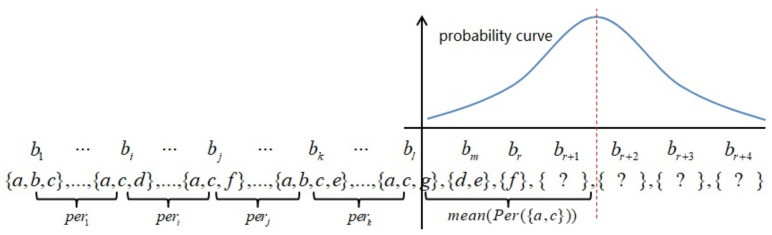
Probability model of periodic pattern {a,c}.

According to Definition 5, we define periodic patterns based on the coefficient of variation. If a pattern has a higher coefficient of variation, which means that the standard deviation is too large compared to the mean value, the pattern is not periodic. Otherwise, we classify it as a periodic pattern. Given a periodic pattern, if it occurs at some time, then it is more likely to reoccur after an average period of time. The probability at a time closer to the time after an average period later has a higher value; otherwise, it has a lower value. We suppose that the probability model of periods follows a normal distribution and has two parameters: mean(Per(p)) and std(Per(p)), as Figure 2 shows.

#### 4.2.2. Principle of Periodic Pattern Prediction

Given transaction sequence $B=\langle b_{1},...,b_{r}\rangle$, and a periodic pattern *p* of *B*, the prediction of the probability of $P(p\subseteq b_{r+1})$ is analogous to the sequential rule prediction.

### 4.3. Association Rule Prediction

Given an association rule $p_{1}\to p_{2}:Conf$, if itemset pattern $p_{1}\subseteq b$, then itemset pattern $p_{2}\subseteq b$ with a probability of $P(p_{2}\subseteq b|p_{1}\subseteq b)=Conf$. If $p_{1}⊈b$, then $P(p_{1}\subseteq b)=0$, and $P(p_{2}\subseteq b|p_{1}\subseteq b)=0$.

After sequential rule prediction and periodic pattern prediction, we obtain a set of candidate products with their probability in $b_{r+1}$. At the same time, we define the probability of $p_{1}\subseteq b_{r+1}$ as $P\left(p_{1}\subseteq b_{r+1}\right)=min\left\{P\left(i\right)|\forall i\in p_{1},\left(i:P\left(i\right)\right)\in pl\right\}$. Therefore, we get the probability of $p_{2}\subseteq b_{r+1}$ as $P\left(p_{2}\subseteq b_{r+1}\right)=P\left(p_{1}\subseteq b_{r+1}\right)\times Conf$. See Algorithm 3.

After pattern prediction, if a product has not been predicted, then it will have a default probability value of zero and is not included in $b_{r+1}$ to be recommended in the pattern prediction stage. Therefore, we obtain a set of candidate products along with their probabilities in the next basket $b_{r+1}$. In $b_{r+1}$, a product with a higher probability will have priority to be recommended to customers. If $|b_{r+1}|<k$, that is, the number of candidate items is less than *k* in $b_{r+1}$, then we will make a preference prediction to continue to select products to recommend until we get all *k* products.
**Algorithm 3:** AssociationRulePrediction$\left(RuleSet,b_{r+1},pl\right)$.
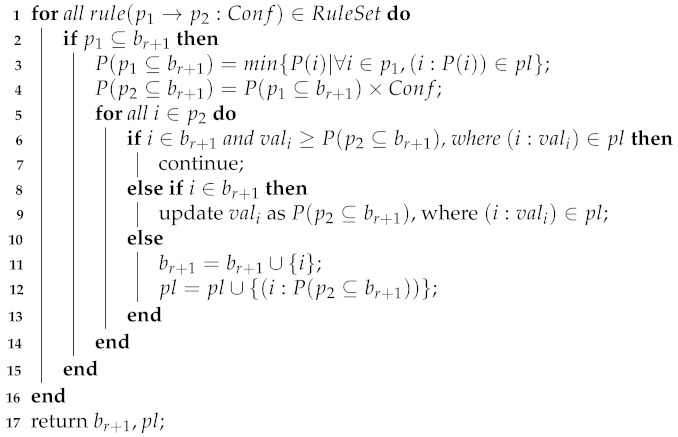


### 4.4. Preference Prediction

In pattern prediction, we selected a set of products to recommend to a customer. If the number of selected products is less than *k*, then we continue to select based on preference prediction.

In preference prediction, if a product is more frequently bought by a customer, then we conclude that the customer has a preference for this product. However, the preference will evolve over time. The purchase distribution of a product over a shopping history will indicate a change in preference. If a product is more frequently bought in the recent baskets than the earlier ones of a customer, then the customer is more and more inclined toward the product; otherwise, if a product is more frequently bought in earlier baskets than recent ones, then the customer tends to be increasingly estranged from the product. According to Definition 8, tendency reflects such a fact. The preference prediction is based on the frequency and tendency of a product in customers’ shopping histories. We first select the products that are more frequent to recommend. If some products have the same frequency, then the product that has a higher tendency is prioritized.

### 4.5. A Comprehensive Example

For the transaction sequence given in Table 1, itemset p={c,f} has an occurrence list of Occur(p)={1,4,8,12,16} and a period list of Per(p)={3,4,4,4}. By calculating, we get mean(Per(p))=3.75 and std(Per(p))=0.866, Coefva(p)=std(Per(p))/mean(Per(p))=0.23. If we set θ=5 and δ=0.5, then *p* is a periodic pattern and its period follows per∼N(3.75,0.866). Now let us predict $b_{17}$ and select 5 products to recommend, viz. k=5. Suppose we have a sequential rule of Example 2 and an association rule of {c,f,g}→{d}:0.9 at the same time. First, in sequential rule prediction, we have $P(\left\{f,g\right\}\subseteq b_{17})=6/21=0.2857$, pl={(f:0.2857),(g:0.2857)}. Second, we use periodic pattern p={c,f} to predict. As Occur(p)={1,4,8,12,16}, λ=16. If $p\subseteq b_{17}$, then $per_{5}=17-16=1$. We have P{per=1}=0.0046 from the probability model. Merging {(c:0.0046),(f:0.0046)} into pl, we get pl={(c:0.0046),(f:0.2857),(g:0.2857)}. Third, the association rule is used to predict. By definition, we have $P(\left\{c,f,g\right\}\subseteq b_{17})=min\left\{0.0046,0.2857,0.2857\right\}=0.0046$, then P({d}$\subseteq b_{17})=0.0046\times 0.9=0.0041$. Finally, the products are selected in the order of 〈f,g,c,d〉. However, there are not enough products to recommend. Thus, we will have a preference prediction.

In preference prediction, all products that are in the individual shopping history, excluding selected products, are sorted by frequency. We have Freq({a})=9, Freq({b})=9, Freq({h})=6 and Freq({e})=5. Because the frequencies of items *a* and *b* are the same, their tendencies are calculated. Item *a* has an occurrence list of Occur({a})={1,3,5,7,8,9,11,13,15}, and item *b* has Occur({b})={1,2,3,7,9,10,11,13,14}. We get Ten({a})=8 and Ten({b})=7.78, and select *a*. Finally, all 5 products are selected.

### 4.6. Relation of Two Prediction Strategies

In pattern prediction, we must set a value to the frequency threshold, confidence threshold and threshold for the coefficient of variation in pattern mining. These parameters determine the weight of pattern prediction and preference prediction.

The confidence threshold parameter is set for sequential rule mining and association rule mining. A higher value of the confidence threshold is set, and fewer sequential rules and association rules are found. The threshold parameter for the coefficient of variation is set for periodic pattern mining. If it has a lower value, then fewer periodic patterns are found. The frequency threshold parameter is set for all three types of patterns. If a higher value is set, then fewer patterns are found. There is no parameter for preference prediction.

In general, if we have a higher value on the frequency threshold and confidence threshold and a lower value on the threshold for the coefficient of variation, then we will select fewer products in pattern prediction. Preference prediction will have a higher weight on the selected result, and pattern prediction will have the opposite effect. Conversely, preference prediction will have a lower weight on the selected result.

## 5. Implementation

### 5.1. Optimizations

In pattern mining, the result grows exponentially as the pattern expands in size. It is a severe problem that traditional pattern mining approaches face to efficiently mine large patterns in dense datasets. The growth of the number of patterns is not proportional to the improvement of prediction performance in our method but leads to heavier workload. In the pattern prediction of our method, if an item is contained in several patterns, it will probably be repeatedly predicted several times, leading to redundancies in workload. The larger the number of patterns or the larger the size of the pattern, the more redundancies in the workload.

For the two reasons mentioned above, we use simple association rules, simple sequential rules and simple periodic patterns to implement pattern prediction.

**Definition** **9.** *(Simple Association Rule) Given an association rule* $p_{1}\to p_{2}:Conf$ *, if both* $p_{1}$ *and* $p_{2}$ *are single item patterns, then we call it a simple association rule.*

**Definition** **10.** *(Simple Sequential Rule) Given a sequential rule* $s_{1}\to s_{2}:Conf$ *, if both* $s_{1}$ *and* $s_{2}$ *are single item patterns, then we call it a simple sequence rule.*

**Definition** **11.** *(Simple Periodic Pattern) Given a periodic pattern p, if p is a single item pattern, then we call it a simple periodic pattern.*

This strategy dramatically reduces the number of patterns and is easier to implement. As a result, our algorithm is incomplete. We use two ready-made algorithms introduced by Philippe Fournier-Viger et al. [13], namely, FPGrowth and RuleGen, to mine simple association rules and simple sequential rules, respectively. The implementation of simple periodic pattern mining will be discussed in the next subsection.

### 5.2. Data Structure

**Figure 3:**
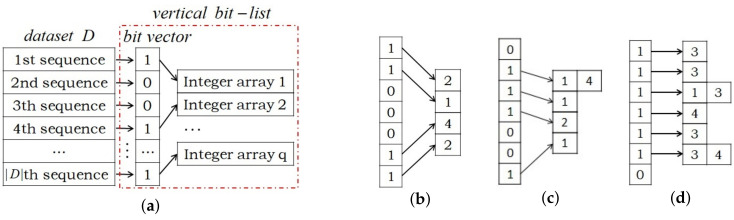
(**a**) shows the verticalbit-list of item i∈I, where $q=|\{B_{c}|\forall B_{c}\in D,\exists b\in B_{c},$i∈b}|. (**b**–**d**) show verticalbit-list of items *b*, *d* and *g*, respectively, in dataset of Table 2.

Inspired by Fabio Fumarola et al. [49], we propose a new data structure named verticalbit-list to represent the dataset. Each item i∈I is assigned a verticalbit-list. A verticalbit-list is made up of a bit vector and several integer arrays, as Figure 3 shows. Bit vectors have a size of the dataset size, i.e. the number of sequences in the dataset. The *j*th bit, j∈{1,...,|D|}, in each bit vector correspond to the *j*th sequence in the dataset *D*. If the *j*th bit in the bit vector of a verticalbit-list is flipped, then means there exist an itemset in the *j*th sequence contains the item to which the verticalbit-list is assigned, and each flipped bit is assigned an integer array to preserve the occurrence list of the item in the sequence to which the flipped bit correspond. If the *j*th sequence contains item *i*, that is, there exist itemsets in the *j*th sequence containing item *i*, then the frequency and tendency of *i* in the *j*th sequence can be calculated by its corresponding integer array, and we can determine whether the item *i* is a simple periodic pattern in the *j*th sequence based on the integer array according to Definition 5, since simple periodic pattern mining is free from pattern growth.

### 5.3. Complexity

In the stage of pattern mining, we dramatically reduce the complexity of our algorithm by mining only simple sequential rules, simple association rules and simple periodic patterns. We mine simple sequential rules and simple association rules in $O(m^{2})$, where *m* is the number of distinct items in I since the rule of two types only contains two items. Simple periodic pattern mining and tendency calculation have a complexity of O(n×v), *n* is the number of all customers and *v* is the average count of products the customers have bought, that is, one scan over the dataset, and the complexity of the prediction stage is the same.

## 6. Experiment

To evaluate the performance of our SPAP algorithm, experiments were conducted on four read-world datasets. First, we assessed the influences of parameters on the weight of pattern prediction and preference prediction. Second, we compared our algorithm with those of the baseline methods and state-of-the-art methods in the evaluation metrics of F1-Score and Hit-Ratio. The experiments were conducted on a computer with an Intel Core I7-8550U 1.8 GHz processor and 8 GB of RAM, running Windows 10 (64-bit version). The SPAP is implemented in Java.

### 6.1. Datasets

We performed our experiments on four real-world transaction sequence datasets. Three of the datasets, Ta-Feng (https://www.kaggle.com/chiranjivdas09/ta-feng-grocery-dataset (Accessed data: 19 October 2021)), Dunnhumby (https://www.kaggle.com/frtgnn/dunnhumby-the-complete-journey (Accessed data: 19 October 2021)) and X5-Retail-Hero (https://www.kaggle.com/mvyurchenko/x5-retail-hero (Accessed data: 19 October 2021)), are based on physical markets; and one dataset, T-Mall (Provided by Guidotti et al. [50] at https://github.com/riccotti/CustomerTemporalRegularities/tree/master/datasets (Accessed data: 19 October 2021)), is based on an online market.

• Ta-Feng is a dataset of a physical market, covering food, stationery and furniture, with a total of 23,812 different items in China. It contains 817,741 transactions made by 32,266 customers over 4 months.
• The Dunnhumby (Dunnh for short) dataset contains household level transactions over two years from a group of 2500 households who are frequent shoppers at a retailer.
• The X5-Retail-Hero (X5RH for short) dataset contains 183,258 transactions made by 9305 customers over 4 months and a total of 27,766 distinct items.
• The T-Mall dataset records four months of online transactions of an online e-commerce website. It contains 4298 transactions belonging to 884 users and 9531 distinct brands considered as items.

In preprocessing these datasets, we remove customers who have fewer than 10 baskets for Ta-Feng, Dunnh and T-Mall, and remove customers who have fewer than 21 baskets for X5RH. For simplicity, we adopt the index of the basket in sequence as the time unit rather than the real date. Table 3 shows the details of these datasets used in our experiment.

### 6.2. Evaluate Metrics F1-Score and Hit-Ratio

Following Guidotti et al. [44], first, we sort the transactions by the timestamps for each customer. Then, we split the dataset into training set and testing set. Testing set contains the latest transaction of all customers for model evaluation. Training set contains the remainder of the transactions of all customers for model training. This is known as leave-one-out strategy. The product set that customer *c* actually buys is denoted as $b_{c}^{\prime }$. The product set recommended to customer *c* is denoted as $b_{c}^{*}$. The metrics we use for evaluation, F1-Score and Hit-Ratio, are defined as

(1) $$\begin{array}{c} precison\left(b_{c}^{\prime },b_{c}^{*}\right)=\frac{|b_{c}^{\prime }\cap b_{c}^{*}|}{|b_{c}^{*}|} \end{array}$$

(2) $$\begin{array}{c} recall\left(b_{c}^{\prime },b_{c}^{*}\right)=\frac{|b_{c}^{\prime }\cap b_{c}^{*}|}{|b_{c}^{\prime }|} \end{array}$$

(3) $$\begin{array}{c} F1-Score=\frac{2\times precison\left(b_{c}^{\prime },b_{c}^{*}\right)\times recall\left(b_{c}^{\prime },b_{c}^{*}\right)}{precison\left(b_{c}^{\prime },b_{c}^{*}\right)+recall\left(b_{c}^{\prime },b_{c}^{*}\right)} \end{array}$$

(4) $$\begin{array}{c} Hit-Ratio=\frac{\sum_{c\in C}I\left(b_{c}^{\prime }\cap b_{c}^{*}\neq ⌀\right)}{\left|C\right|} \end{array}$$

where I(*) is an indicator function. The F1-Score is reported by the average value of all customers.

Furthermore, to evaluate the contribution of two prediction strategies, we introduce a new measure: Weight. Let $b_{pa}$ denote all the items selected in pattern prediction for all customers, and $b_{pr}$ denote all the items selected in preference prediction for all customers. The number of all items selected to recommend to all customers is $\sum_{c\in C}\left|b_{c}^{*}\right|$, and we have $|b_{pa}\left|+\right|b_{pr}|=\sum_{c\in C}\left|b_{c}^{*}\right|$. The weights of pattern prediction and preference prediction are denoted as $Weight_{pa}$ and $Weight_{pr}$, respectively, and defined as

(5) $$\begin{array}{c} Weight_{pa}=\frac{|b_{pa}|}{\sum_{c\in C}\left|b_{c}^{*}\right|} \end{array}$$

(6) $$\begin{array}{c} Weight_{pr}=\frac{|b_{pr}|}{\sum_{c\in C}\left|b_{c}^{*}\right|}=1-Weight_{pa} \end{array}$$

due to the complementation of $Weight_{pa}$ and $Weight_{pr}$, we report $Weight_{pa}$ only. A higher value of $Weight_{pa}$ means that more items are selected in pattern prediction, and vice versa. In the remainder of this paper, we use Weight to replace $Weight_{pa}$. Note that if the number of all distinct products customer *c* has bought is less than *k*, then we cannot select a set of products including more than or equal to *k* items to recommend to him or her, that is, $|b_{c}^{*}|\leq k$, leading to $\sum_{c\in C}\left|b_{c}^{*}\right|\leq k\times n$, where |C|=n.

### 6.3. Influence of Parameters

Our algorithm is composed of two prediction strategies. We obtain the best performance only on the right proportion of weight on pattern prediction and preference prediction. There are four parameters in pattern prediction: relative frequency threshold $\theta_{r}$ and confidence threshold η for sequential rule mining and association rule mining, threshold for coefficient of variation δ and absolute frequency threshold $\theta_{p}$ for periodic pattern mining. All of these parameters have an influence on the weight of these two prediction strategies. In this subsection, we will evaluate the influences of these parameters. *k* is set to the average basket size of each dataset. The values for the remainder of the parameters are preset to be $\theta_{r}=0.16$, η=0.2, δ=0.2 and $\theta_{p}=5$ for Ta-Feng, $\theta_{r}=0.35$, η=0.7, δ=0.4 and $\theta_{p}=12$ for Dunnh, $\theta_{r}=0.15$, η=0.4, δ=0.8 and $\theta_{p}=10$ for X5RH, and $\theta_{r}=0.1$, η=0.4, δ=0.8 and $\theta_{p}=5$ for T-Mall.

**Figure 4:**
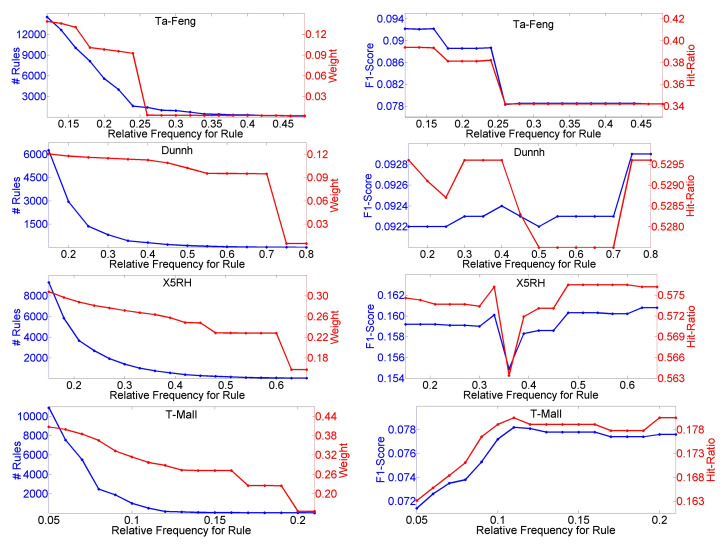
Influences of different values of $\theta_{r}$ on number of rules, Weight, F1-Score and Hit-Ratio.

**Figure 5:**
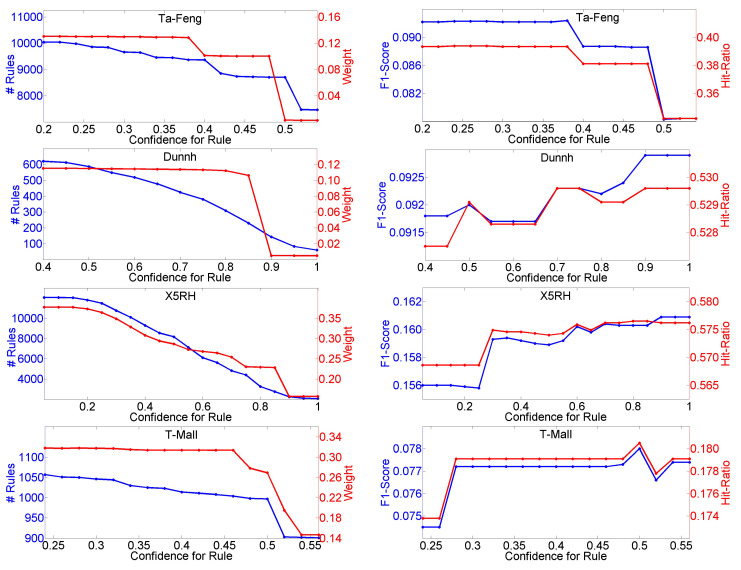
Influences of different values of η on number of rules, Weight, F1-Score and Hit-Ratio.

First, we test the parameter of relative frequency threshold $\theta_{r}$, and the results are shown in Figure 4. We can see that when $\theta_{r}$ rises, the total number of distinct rules, including sequential rules and association rules, and Weight decrease in all cases of the four datasets. Weight is always lower than 0.5, which means that preference prediction plays a dominant role. For the Ta-Feng dataset, when $\theta_{r}$ has a value less than 0.16, both F1-Score and Hit-Ratio remain unchanged and achieve the optimal values, and the situation is the same on the Dunnh dataset when $\theta_{r}$ is greater than 0.75. Both F1-Score and Hit-Ratio are constant on the X5RH dataset when $\theta_{r}$ is greater than 0.63. For the T-Mall dataset, when $\theta_{r}$ is near to 0.11, both F1-Score and Hit-Ratio achieve the optimal values. Figure 5 shows the influences of different values for threshold η. As η rises, the number of rules and Weight decrease on all four datasets. Both F1-Score and Hit-Ratio achieve the optimal values on the Ta-Feng dataset when η is less than 0.38, achieve the optimal values on the Dunnh when η is greater than 0.9 and achieve the optimal values on the T-Mall dataset when η is equal to 0.5, respectively. When η is greater than 0.8, F1-Score or Hit-Ratio achieves the optimal value on the X5RH dataset.

**Figure 6:**
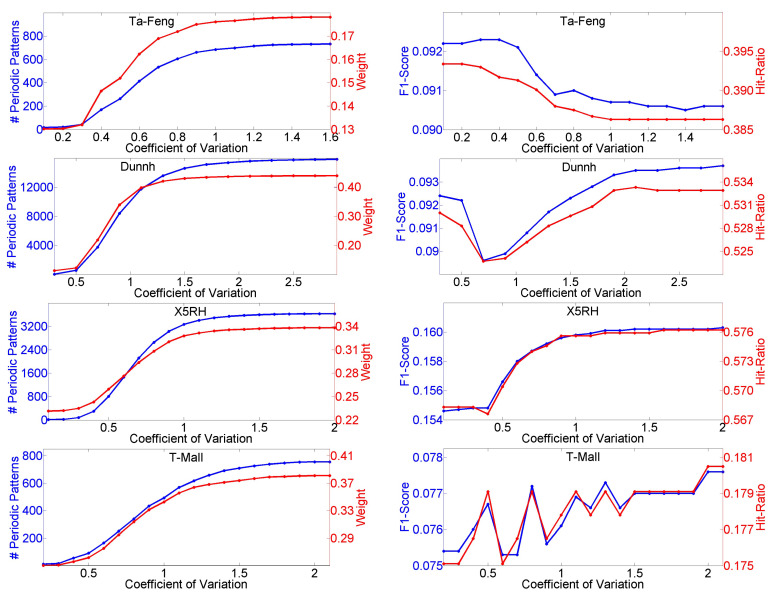
Influences of different values of δ on number of periodic patterns, Weight, F1-Score and Hit-Ratio.

The threshold for the coefficient of variation δ is set for periodic pattern mining. A higher value for δ will lead to a larger number of periodic patterns, as Figure 6 shows. At the same time, a larger number of periodic patterns results in a higher Weight on all four datasets. When δ is less than 0.4, F1-Score or Hit-Ratio achieves the optimal value on the Ta-Feng dataset. Both F1-Score and Hit-Ratio remain unchanged and achieve the optimal values on the Dunnh dataset when δ is greater than 2.8, and the situation is the same on the X5RH and T-Mall datasets when δ is greater than 2.0.

**Figure 7:**
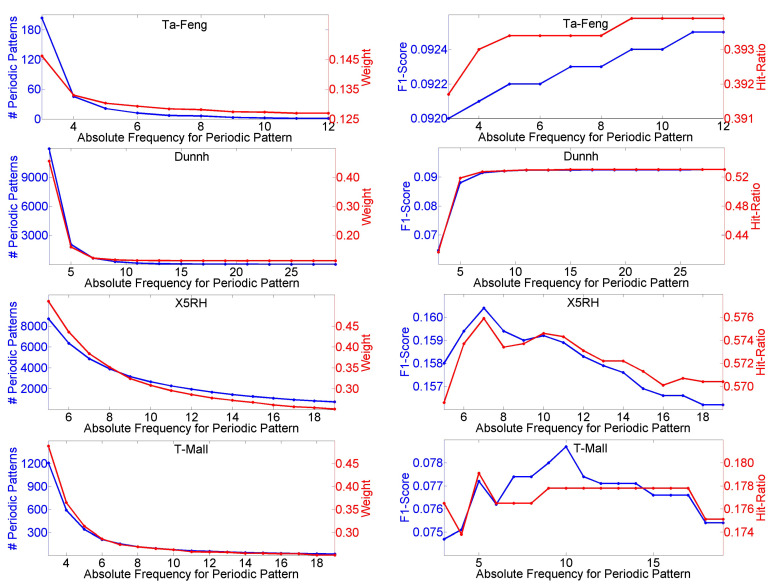
Influences of different values of $\theta_{p}$ on number of periodic patterns, Weight, F1-Score and Hit-Ratio.

Finally, we test the absolute frequency threshold $\theta_{p}$ for periodic pattern mining. As shown in Figure 7, when $\theta_{p}$ rises, the number of periodic patterns and Weight decrease on all four datasets. Both F1-Score and Hit-Ratio remain unchanged and achieve the optimal values on the Ta-Feng dataset when $\theta_{p}$ is greater than 11, and the situation is the same on the Dunnh dataset when $\theta_{p}$ is greater than 27. In the case of X5RH, when $\theta_{p}$ is equal to 7, both F1-Score and Hit-Ratio achieve the optimal values. For the T-Mall dataset, F1-Score and Hit-Ratio achieve their optimal values when $\theta_{p}$ is equal to 10 and 5, respectively.

### 6.4. Comparison with Baseline Methods and State-of-the-Art Methods

In this subsection, we report the comparisons of our method with baseline methods, including TOP, MC, CLF and NMF; and state-of-the-art methods, including HRM, TBP, TIFU and UPCF.

• TOP predicts the top-k most frequent items with respect to their appearance, i.e., the number of times that they are purchased, in a customer’s purchasing history $B_{c}$.
• MC [40] makes the prediction based on the last purchase $b_{r_{c}}$ and on a Markov chain calculated on $B_{c}$.
• CLF [40]: Due to space limitations, we do not discuss here. See [40] for more details.
• NMF (Non-negative Matrix Factorization) [51] is a collaborative filtering method that applies a non-negative matrix factorization to the customers-items matrix. The matrix is constructed from the purchase history of all customers.
• HRM (Hierarchical Representation Model) [41] employs a two-layer structure to construct a hybrid representation over customers and items purchase history *B* from last transactions: the first layer represents the transactions by aggregating item vectors from the last transactions, while the second layer realizes the hybrid representation by aggregating the user’s vectors and the transactions representations.
• TBP [44] is a new pattern-based method proposed by Guidotti et al. [44] that seeks to simultaneously capture the co-occurrence, sequentiality, periodicity and recurrence of the items in basket sequences.
• TIFU [47] (https://github.com/HaojiHu/TIFUKNN (Accessed data: 19 October 2021)) is a k-nearest neighbors (kNN) based method.
• UPCF [48] (https://github.com/MayloIFERR/RACF (Accessed data: 19 October 2021)) denotes User Popularity-based Collaborative Filtering. The model considers a user-based collaborative approach that relies on similar users to find new items that can be of interest to the target user.

**Figure 8:**
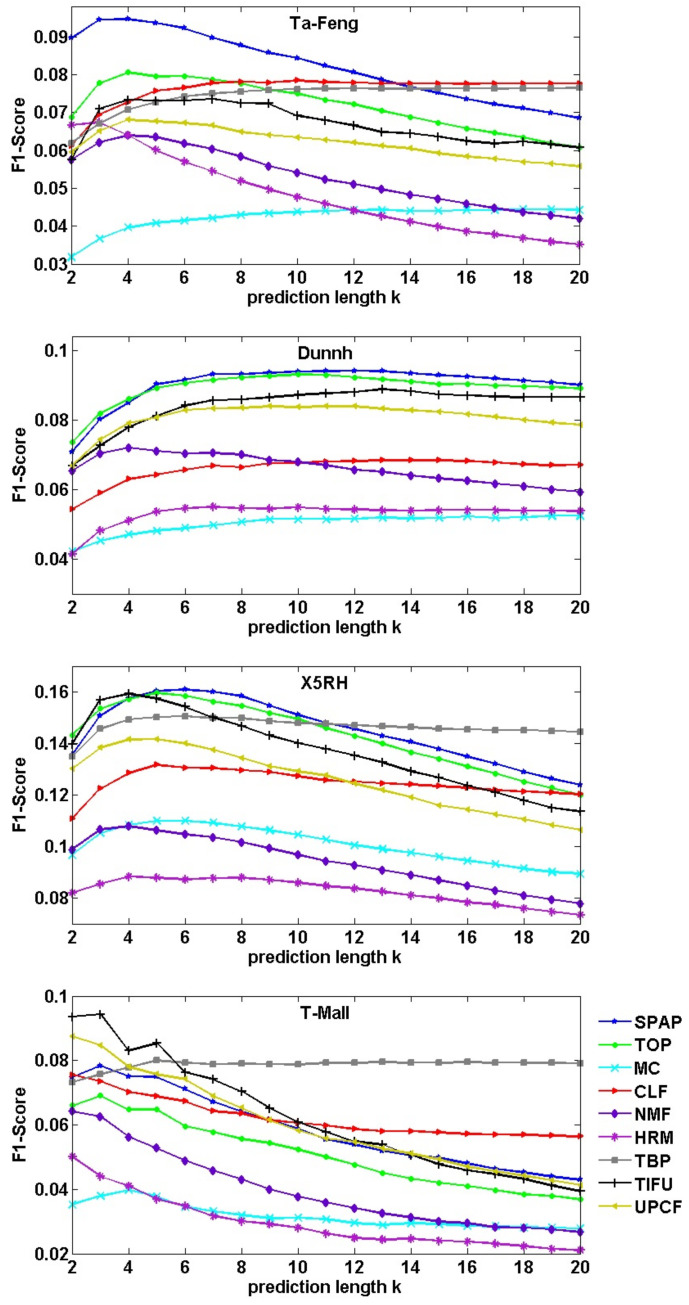
Comparison of F1-Score with those of the baseline methods and state-of-the-art methods under different values of *k*.

**Figure 9:**
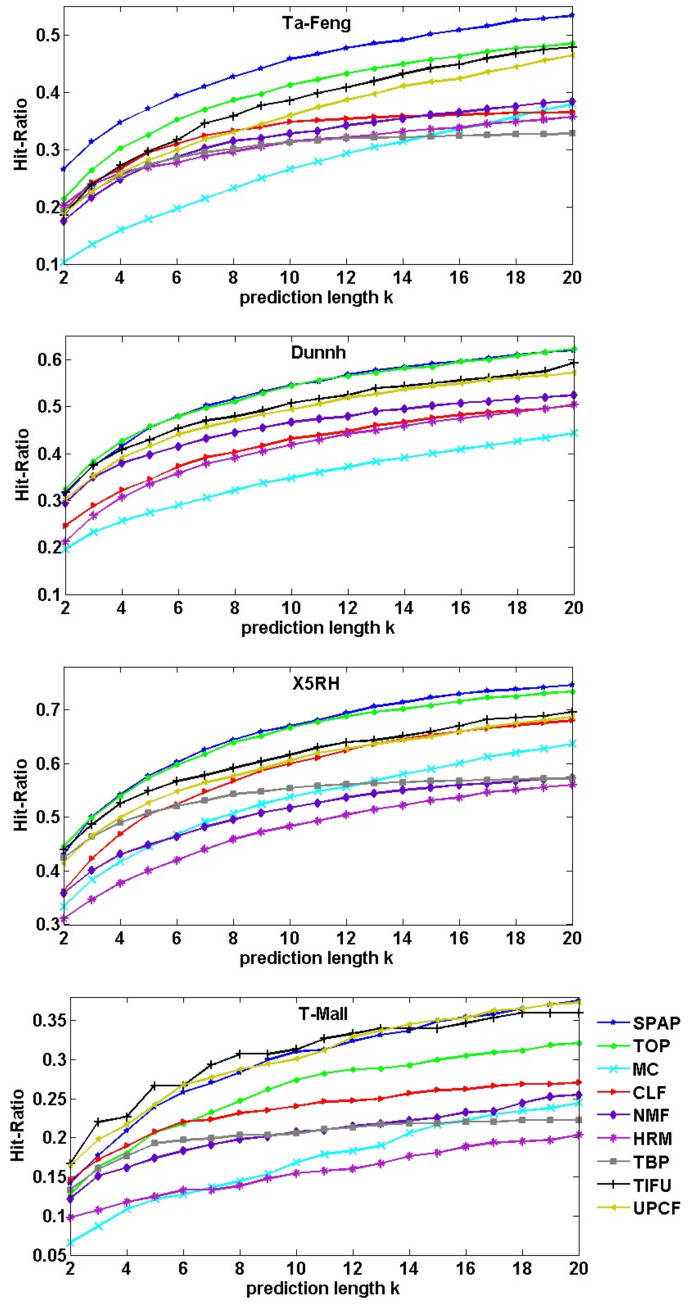
Comparison of Hit-Ratio with those of the baseline methods and state-of-the-art methods under different values of *k*.

Source code for MC, CLF, NMF, HRM and TBP are provided by [44] (https://github.com/GiulioRossetti/tbp-next-basket (Accessed data: 19 October 2021)); and all the algorithms run under the recommended parameter values. Results are shown in Figure 8 and Figure 9. TBP runs overtime on the Dunnh dataset.

Figure 8 shows the results of F1-Score, we can see that our SPAP algorithm has the best F1-Score on the Ta-Feng dataset when *k* is less than 14, and closes to TOP on the Dunnh and X5RH datasets. Figure 9 shows the results of Hit-Ratio, SPAP outperforms the other algorithms on the Ta-Feng dataset, and closes to TOP on the Dunnh and X5RH datasets.

We noticed that all algorithms, except TBP, exhibit poor performance for F1-Score when *k* is too higher or lower than the average basket size. The most obvious finding is on T-Mall dataset, which has the smallest average basket size. Because F1-Score is relevant to the size of recommended basket $b_{c}^{*}$. A higher value for *k* means we will select more products to recommend, leading to a large size for $b_{c}^{*}$ and a lower value for $precison(b_{c}^{\prime },b_{c}^{*})$; otherwise, we have a lower value for $recall(b_{c}^{\prime },b_{c}^{*})$. However, when the value of *k* increases, it results in a higher possibility of being hit, and Hit-Ratio rises naturally. Only *k* has a value nearer to the average basket size, and we have a fairer comparison.

As described above, almost all algorithms exhibited their best performance when *k* was set as a value of the average basket size. Table 4 lists the performance results in which *k* has a value of the average basket size, k=6, k=9, k=5 and k=3 for Ta-Feng, Dunnh, X5RH and T-Mall, respectively. TIFU has the best performances on the T-Mall dataset. However, our SPAP algorithm outperforms other algorithms on the Ta-Feng, Dunnh and X5RH datasets.

Patterns have a virtue of reflecting customers’ shopping habits. Due to the fact that people visit physical stores more regularly than online stores, our pattern-based model SPAP achieves the best performances on physical store datasets Ta-Feng, Dunnh and X5RH. Table 5 lists the improvements of SPAP compared with TOP on the Ta-Feng, Dunnh and X5RH datasets; and compared with TIFU on the T-Mall dataset.

### 6.5. Running Time

All of these algorithms have a running time ranging from several seconds to several hours. TOP is always the fast one [44]. In this subsection, we compare the running time (training time and prediction time) of our algorithm with TOP. Both of them are implemented in Java. The results are shown in Table 6. Source code for MC, CLF, NMF, HRM, TBP, TIFU and UPCF are implemented in Python, so we do not report their running times here. We terminate the processe of TBP on the Dunnh dataset when it runs over two days.

## 7. Conclusions

In this paper, we propose a pattern-based model for next basket prediction. The method includes pattern prediction and preference prediction. In pattern prediction, first, all sequential rules, periodic patterns and association rules are found together with their statistical characteristics. Then, probability models are built based on their statistical characteristics. Afterward, we use the probability models to calculate the probability of all products in the next basket for a customer. The products that have a higher probability will be selected to recommend to him or her. If *k* products have been selected, then continue to the prediction of the next customer; otherwise, make preference predictions. In preference prediction, the product that is more frequent in the individual shopping records will be selected first. If some products have the same frequency, then the product that has a higher tendency will be selected. Until all *k* products are selected. Experiments show that our algorithm outperforms those of the baseline methods and state-of-the-art methods on three of four real-world transaction sequence datasets.

## Figures and Tables

**Table 1:** Transaction sequence of a customer.

Transa. ID	Timestamp	Basket	Transa. ID	Timestamp	Basket
$b_{1}$	2010-01-01	{a,b,c,f}	$b_{9}$	2010-02-06	{a,b,g,h}
$b_{2}$	2010-01-05	{b,c,d}	$b_{10}$	2010-02-14	{b,c,d}
$b_{3}$	2010-01-07	{a,b,e,f,h}	$b_{11}$	2010-02-19	{a,b,f,h}
$b_{4}$	2010-01-11	{c,d,f,h}	$b_{12}$	2010-02-25	{c,d,f,g,h}
$b_{5}$	2010-01-16	{a,d,e,f,g}	$b_{13}$	2010-03-02	{a,b,c}
$b_{6}$	2010-01-19	{c,e,g,h}	$b_{14}$	2010-03-09	{b,e,g}
$b_{7}$	2010-01-28	{a,b,c,g}	$b_{15}$	2010-03-17	{a,c,d,e}
$b_{8}$	2010-01-31	{a,c,f,g}	$b_{16}$	2010-03-28	{c,f}

**Table 2:** Illustrative transaction sequence database containing seven customers.

Customer ID	Transaction Sequences
$c_{1}$	〈{a,c},{b,e},{f,g}〉
$c_{2}$	〈{b,c,d},{a,c},{f,g},{d,e}〉
$c_{3}$	〈{d,e,g},{a,c},{f,g}〉
$c_{4}$	〈{a,c},{d,e},{c},{f,g}〉
$c_{5}$	〈{a,c},{e},{f,g}〉
$c_{6}$	〈{a,c,e},{a,c},{f,g},{b,f,g}〉
$c_{7}$	〈{d},{a,b,c}〉

**Table 3:** Characteristics of running datasets.

Dataset	♯ Customers	♯ Items	♯ Baskets	Average Basket Size
Ta-Feng	2374	18,138	39,533	5.58
Dunnh	2402	91,779	273,474	9.36
X5RH	2924	22,921	99,357	5.40
T-Mall	748	9436	31,035	2.52

**Table 4:** Comparison with those of the baseline methods and state-of-the-art methods when *k* is set as average basket size value. The bold is the maximum of the all, and the underline is the maximum of the competing algorithms.

	Dataset	SPAP	TOP	MC	CLF	NMF	HRM	TBP	TIFU	UPCF
F1-Score	Ta-Feng (k=6)	**0.0922**	0.0796	0.0415	0.0765	0.0617	0.0570	0.0742	0.0731	0.0672
	Dunnh (k=9)	**0.0935**	0.0926	0.0514	0.0675	0.0685	0.0545	-	0.0864	0.0839
	X5RH (k=5)	**0.1604**	0.1596	0.1100	0.1316	0.1057	0.0879	0.1502	0.1574	0.1417
	T-Mall (k=3)	0.0783	0.0691	0.0379	0.0735	0.0626	0.0440	0.0757	**0.0944**	0.0847
Hit-Ratio	Ta-Feng (k=6)	**0.3934**	0.3517	0.1963	0.3096	0.2894	0.2768	0.2850	0.3158	0.2991
	Dunnh (k=9)	**0.5329**	0.5287	0.3370	0.4165	0.4554	0.4049	-	0.4917	0.4833
	X5RH (k=5)	**0.5771**	0.5728	0.4454	0.5056	0.4457	0.4008	0.5075	0.5486	0.5266
	T-Mall (k=3)	0.1778	0.1631	0.0869	0.1725	0.1457	0.1072	0.1602	**0.2200**	0.1979

**Table 5:** Improvements vs. the maximum of the competing algorithms.

	Improvements vs. TOP			Improvements vs. TIFU
	**Ta-Feng**	**Dunnh**	**X5RH**	**T-Mall**
F1-Score	15.8%	1%	0.5%	−17.1%
Hit-Ratio	11.9%	0.8%	0.8%	−19.2%

**Table 6:** Running times.

	Ta-Feng	Dunnh	X5RH	T-Mall
TOP	0.0476 s	0.444 s	0.192 s	0.078 s
SPAP	0.749 s	12.838 s	2.824 s	0.234 s

## Data Availability

The datasets are available at the following links: https://www.kaggle.com/chiranjivdas09/ta-feng-grocery-dataset (Accessed data: 19 October 2021), https://www.kaggle.com/frtgnn/dunnhumby-the-complete-journey (Accessed data: 19 October 2021), https://www.kaggle.com/mvyurchenko/x5-retail-hero (Accessed data: 19 October 2021), https://github.com/riccotti/CustomerTemporalRegularities/tree/master/datasets (Accessed data: 19 October 2021).
